# Histamine H3 receptor antagonists/inverse agonists on cognitive and motor processes: relevance to Alzheimer's disease, ADHD, schizophrenia, and drug abuse

**DOI:** 10.3389/fnsys.2012.00072

**Published:** 2012-10-23

**Authors:** Divya Vohora, Malay Bhowmik

**Affiliations:** Faculty of Pharmacy, Department of Pharmacology, Jamia Hamdard (Hamdard University)New Delhi, India

**Keywords:** histamine H3 receptor, Alzheimer's disease, ADHD, schizophrenia, drug abuse

## Abstract

Histamine H3 receptor (H3R) antagonists/inverse agonists possess potential to treat diverse disease states of the central nervous system (CNS). Cognitive dysfunction and motor impairments are the hallmark of multifarious neurodegenerative and/or psychiatric disorders. This review presents the various neurobiological/neurochemical evidences available so far following H3R antagonists in the pathophysiology of Alzheimer's disease (AD), attention-deficit hyperactivity disorder (ADHD), schizophrenia, and drug abuse each of which is accompanied by deficits of some aspects of cognitive and/or motor functions. Whether the H3R inverse agonism modulates the neurochemical basis underlying the disease condition or affects only the cognitive/motor component of the disease process is discussed with the aim to provide a rationale for their use in diverse disease states that are interlinked and are accompanied by some common motor, cognitive and attentional deficits.

Histamine H3 receptor (H3R) antagonists/inverse agonists have revealed potential to treat diverse disease states of the central nervous system (CNS) including Alzheimer's disease (AD), attention-deficit hyperactivity syndrome (ADHD), schizophrenia, obesity, pain, epilepsy, narcolepsy, substance abuse, etc. (Leurs et al., 2011; Passani and Blandina, 2011). The histamine H3R was first identified in the rat brain (Arrang et al., 1983), its presence in the human brain was confirmed a few years later (Arrang et al., 1988); and it was successfully cloned and functionally expressed by Lovenberg et al. (1999) following which there was spurt of activities by several academic groups and pharmaceutical companies to develop a flurry of compounds that could therapeutically modulate the functions of this receptor. Though there is no direct pathophysiological mechanism linking any disease condition of the CNS with histamine, the distinct localization of H3Rs in the CNS coupled with the fact that it modulates the release of other neurotransmitters in the brain via its action on heteroreceptors on non-histaminergic neurons led to evaluation of its ligands in various brain diseases (Nuutinen and Panula, 2010).

H3R, a G-protein coupled receptor (GPCR, coupled to Gi/o proteins), is able to signal on its own i.e., without activation by an agonist, and thus displays constitutive activity. H3R activation inhibits synthesis of histamine through adenylate cyclase/protein kinase A and calcium/calmodulin-dependent protein kinase type II (CaMKII) pathways. In addition, it can activate phospholipase A2 (PLA2) mediated release of arachidonic acid (AA) and phosphoinositol-3-kinase activity resulting in activation of the Akt/glycogen synthase kinase (GSK)-3β axis (Leurs et al., 2005). Evidence linking how these signaling pathways relate to the efficacy of H3R antagonists in various disease states is scarce. Though the role of histamine H3Rs as therapeutic target in brain diseases have been the subject of many recent reviews, here we have tried to bring together various neurobiological/neurochemical evidences available so far following H3R ligands in the pathophysiology of AD, ADHD, schizophrenia, and drug abuse each of which is accompanied by deficits of some aspects of cognitive and/or motor functions. Whether the H3R inverse agonism modulates the neurochemical basis underlying the disease condition or affects only the cognitive/motor component of the disease process is discussed.

## Alzheimer's disease

AD is a chronic and progressive neurodegenerative brain disease and one of the most prevalent forms of dementia affecting nearly 20 million people worldwide (Chen et al., 2012). Role of histamine in AD is well documented. But, owing to variable findings concerning the histamine levels in brain compartments of AD patients, a direct correlation between histaminergic neurotransmission and AD pathology cannot be made and hence the rationale of using H3R antagonists in the treatment of AD is rather complex (Fernández-Novoa and Cacabelos, 2001; Brioni et al., 2011). Few studies by a group of Spanish researchers claim that histamine levels in AD patients is increased in areas such as temporal and frontal cortex, basal ganglia, and hippocampus (Cacabelos et al., 1989) also, together with its metabolites, in the cerebrospinal fluid (CSF) and serum of AD patients (Cacabelos et al., 1992; Fernandez-Novoa et al., 1997), although this rise in histamine level has been vaguely attributed to its non-neuronal pool i.e., mast cells in the CNS [reviewed by Fernández-Novoa and Cacabelos (2001)].

On the contrary to the above findings, several independent studies show decline in histamine levels in AD brains. The tuberomamillary nucleus (TMN) seems to be affected in AD as occurrence of high density of neurofibrillary tangles (NFT) surrounding tuberomammillary histaminergic neurons was observed in AD patients (Saper and German 1987), while, the distribution and number of histaminergic cell bodies remained similar to that of normal brains (Airaksinen et al., 1991). Braak et al. (1993) suggested that during the course of AD, diminished histamine production is paralleled by build up of NFT in the TMN in the early stages. In agreement with this, a significant loss of large-sized histamine containing neurons in the rostral TMN was observed where numerous NFT were found, indicative of a central histaminergic dysfunction (Nakamura et al., 1993). Similarly, high performance liquid chromatography results in some studies have shown reduction in histamine content in the hypothalamus, hippocampus, and temporal cortex of AD brains (Mazurkiewicz-Kwilecki and Nsonwah, 1989; Panula et al., 1998). But clearly, the number studies that evidenced a decline in histamine level is more than the studies that observed an excess of brain histamine in AD. These discrepancies may have arisen from the putative confounding factors like post-mortem delays and differences in subjects assessed e.g., control subjects from a different population than AD patients (Panula et al., 1998).

Central histaminergic fibres originating from the TMN in the posterior hypothalamus (Brown et al., 2001) widely projects into different brain areas including the cerebral cortex, thalamus, basal ganglia, amygdala, and hippocampus, where histamine is crucially associated with a large number of basic physiological functions including sensory and motor functions, cognition, attention, learning, and memory (Haas et al., 2008). Blockade of human H3 autoreceptor by thioperamide evokes the increase of the neuronal histamine release (Flik et al., 2011), and the neurotransmitter modulates cognition processes via both human H1 and H2 receptors or via cholinergic and GABAergic interneurons either directly via excitation of neocortical pyramidal neurons and thalamic relay neurons or indirectly via excitation of ascending cholinergic neurons (Bacciottini et al., 2001; Haas and Panula, 2003; Haas et al., 2008). The H3R heterogeneity was revealed in a microdialysis study, where local TMN administration of H3R antagonist yielded variable histamine release in different brain areas: whereas the concentration increased in TMN, nucleus basalis magnocellularis (NBM), and prefrontal cortex (PFC), it remained unchanged in striatum and nucleus accumbens (NAc). There was differential regulation of neurotransmitter release in a region-specific manner in the brain (Giannoni et al., 2010). The degeneration of histamine neurons in AD does not parallel with the extent of H3R expression. In fact, only a very minor portion of brain H3Rs are located in histaminergic neurons and are largely expressed in deep cortical and thalamocortical projection neurons and in striatal neurons among many other neuron populations. Thus, despite a strong AD related neurodegeneration of the TMN in its severe late stages, H3R densities are either preserved in the brains of amyloid overexpressing TASTPM transgenic mice as well as of AD patients as revealed by receptor binding data (Medhurst et al., 2007, 2009) or increased expression of H3R mRNA (in brains of female AD patients) is evidenced by PCR studies (Shan et al., 2012), signifying that they constitute an adequate target to improve the cognitive disorders encountered in AD.

Recently H3R ligands are being extensively investigated for their potential as a therapeutic agent for cognitive deficits (Sander et al., 2008; Tiligada et al., 2009; Brioni et al., 2011; Leurs et al., 2011). In preclinical studies, H3R antagonists enhance histamine neuron activity and a simultaneous improvement in cognition and learning was observed (Passani et al., 2004). Clearly, the promising preclinical findings have given strong impetus for numerous pharmaceutical companies to embark on proof-of-concept clinical studies with a number of H3 antagonists for diverse neurological conditions including AD (Drahl, 2009; Abbott-press release, 2011; GSK-pipeline, 2011). Recently phase II trials of H3R antagonists ABT-288 (NCT-ID NCT01018875), GSK239512 (NCT-ID NCT01009255), and MK0249 (NCT-ID NCT00420420) for mild to moderate AD have been completed. The outcomes of these trials have not yet been publicly divulged. Another compound PF-03654746 has completed phase I clinical trial (NCT-ID NCT01028911) to evaluate its safety, tolerability and blood levels in mild to moderate AD patients and is subjected to phase II trial (http://www.pfizer.com/files/research/pipeline/2010_0927/pipeline_2010_0927.pdf).

Degeneration of the basal forebrain cholinergic neurons occurs early in the course of AD and has been correlated well with the observed cognitive decline in such patients (Coyle et al., 1983). Reduced acetylcholine (ACh) levels and function in the brain is considered to be the classical attribute to cognitive deficits in AD. Researchers have found that besides controlling histamine release, H3R antagonists may alleviate AD associated cognitive deficits by augmenting release of other neurotransmitters including ACh (Passani et al., 2004; Haas et al., 2008). H3R antagonists enhanced ACh levels in the PFC and in the dorsal hippocampus accompanied by an improvement of cognitive functions in behavioral studies in rodents (Fox et al., 2005). However, the progressive cholinergic cell loss in basal forebrain linked with AD (Whitehouse et al., 1982) probably confines the therapeutic efficacy of drugs to be reliant on endogenous ACh synthesis and doesn't provide disease-modifying efficacy in addition to symptomatic improvement.

The two cardinal features of AD pathology speculated to be the potential targets of disease-modifying drugs are β-amyloid (Aβ), a product of aberrant amyloid precursor protein (APP) leading to production of extracellular Aβ plaques, and NFT arising from hyperphosphorylation of tau, a microtubule-associated protein (Giacobini and Becker, 2007). Activation of cellular pathways that inhibit tau kinase signaling and subsequent tau hyperphosphorylation is considered to be the most feasible strategy to prevent tau aggregation and associated pathological effects. Suppression of tau protein can also block Aβ-induced apoptosis thereby reducing cognitive deficits (Martin et al., 2011). A recent study using rat PC12 tumour cell line demonstrated neuroprotective effects of clobenpropit, an H3R antagonist, on injury produced by Aβ42 toxicity (Fu et al., 2010). Though there are several mechanisms that are thought to be involved in Aβ toxicity, a glutamate mediated mechanism was suggested in this study. An encouraging preclinical evidence with the H3R antagonist ABT-239 suggests that H3R antagonism might not only deliver symptomatic treatment in AD, but also possess disease-modifying benefits (Bitner et al., 2011). Acute administration of the H3R antagonist ABT-239 in CD1 mice increased cortical CREB (cAMP response element binding protein) and Ser9-GSK-3β phosphorylation (GSK-3β phosphorylated at serine 9) at cognitive enhancing doses. Furthermore, donepezil (an acetylcholinesterase inhibitor) at clinical doses induced CREB phosphorylation consistent with a pro-cognitive action, but unlike ABT-239, did not alter Ser9-GSK3β level (Bitner et al., 2011). Together, these findings indicated that increased Ser9-GSK3β phosphorylation induced by ABT-239 is independent of increased ACh release. In an earlier study by the same group, both CREB and Ser9-GSK3β phosphorylation were shown to be down-regulated in the Tg2576 (APP/Aβ) transgenic mouse model of AD (Bitner et al., 2009). However, a 2-week infusion of ABT-239 in Tg2576 mice restored reduced cortical CREB and hippocampal pSer9-GSK3β phosphorylation. In parallel studies conducted in female TAPP mice, an AD transgenic line expressing both APP and tau transgenes, ABT-239 infusion reversed tau hyperphosphorylation in the spinal cord and hippocampus (Bitner et al., 2011). Mechanistically, ABT-239 produced pSer9-GSK3β changes in α7 nicotinic ACh receptor (nAChR) knockout mice, an effect also observed in normal mice that exhibit α7 nAChR agonist-induced phosphorylation (Bitner et al., 2009), suggesting that H3R antagonist-mediated signaling (increased pSer9-GSK3β) does not appear to require ACh-stimulated α7 nAChR activation (Bitner et al., 2011). These *in vivo* signaling studies boost the exciting prospect that H3R antagonists activate multiple signaling pathways that may translate into improved disease-modifying efficacy in patients with AD, along with symptomatic alleviation (Bitner et al., 2011; Brioni et al., 2011). Thus, it can be hypothesized that H3R antagonist-evoked neurotransmitter release (e.g., ACh) leads to activation of postsynaptic receptor pathways such as phosphorylation-activation of CREB, a transcription factor relevant to cognitive function, and phosphorylation of inhibitory residue Ser9 of GSK3β, a primary tau kinase in AD responsible for tau hyperphosphorylation (Hooper et al., 2008; Bitner et al., 2011). This, together with the disease-modifying capacity of H3R antagonist might also impact the underlying disease pathology (e.g., tau phosphorylation) beyond mere symptomatic alleviation (reviewed by Brioni et al., 2011). In line with the above view, Abbott has suggested a combinatorial treatment of cognitive disorders consisting of a nAChR ligand (either α4β2 or α7 subtype) and a H3R antagonist e.g., ABT-239 (Abbott laboratories, WO2009082698; 2009) which can also include psychostimulants (e.g., methylphenidate) or monoamine re-uptake inhibitors (e.g., atomoxetine) to achieve greater clinical efficacy (Lazewska and Kiec-Kononowicz, 2010).

## Attention-deficit hyperactivity disorder

ADHD is a disorder most prevalent in children characterized by persistent carelessness, hyperactivity, and impulsivity. The current pharmacological treatments of ADHD include stimulants (methylphenidate, amphetamines, etc.), non-stimulant (atomoxetine), α2 agonists (clonidine and guanfacine) etc. However, these treatments (mainly stimulants) are associated with significant adverse effects and abuse liability. The potential usefulness of H3R antagonists in this pathology is buttressed by their pro-attentional and pro-cognitive activity in a number of rodent models [such as object recognition task, social recognition task, spontaneous hypertensive rats (SHR), and five-choice stimulus reaction time test (5-CSRTT)] which is devoid of any psychomotor activation and abuse liability (Gemkow et al., 2009; Kuhne et al., 2011; Passani and Blandina, 2011). ADHD involves interplay of multiple neurotransmitter systems mainly of dopaminergic and noradrenergic systems but also of cholinergic and serotonergic systems (Curatolo et al., 2009; Cortese, 2012). While stimulants block the reuptake of dopamine (DA) and norepinephrine (NE) into presynaptic neuron (amphetamine in addition also promotes release), atomoxetine, a non-stimulant drug, blocks NE transporter thereby increasing concentrations of NE throughout the brain but DA only in PFC (Cortese, 2012). In agreement, H3R antagonists have been shown to elevate the release of neurotransmitters involved in cognition e.g., ACh and DA in the PFC (Fox et al., 2005; Ligneau et al., 2007), ACh, DA, and NE in the anterior cingulate cortex (Medhurst et al., 2007; Southam et al., 2009), and AChh in the hippocampus (Fox et al., 2005).

In preclinical models, pharmacological alterations that antagonize the cholinergic system or enhance the various neurotransmitter systems like DA, orexin, cannabinoids systems including histamine cause hyperactivity [an increase in locomotor activity (LA)] that accompanies various neurological disorders including ADHD The LA can be decreased by genetic alterations that reduce the level of histamine (e.g., in HDC KO mice) or by lesions of the TMN (Viggiano, 2008). Recently, H3R antagonist (carnicine, a stable analog of the naturally occurring dipeptide carnosine) attenuated hyperlocomotion in an ADHD-specific model with neonatal habenula lesion without having an effect on attention-deficit (Goto and Lee, 2011). In other studies, antagonists of H3R have demonstrated pro-attentional effects in various ADHD-specific animal models including five-trial inhibitory avoidance in SHR pups (thioperamide, ABT-239, GT-2331, and ciproxifan) (Fox et al., 2002; Komater et al., 2003) and impairment in a 5-CSRTT (ciproxifan) (Day et al., 2007). In addition, CEP-26401 (irdabisant), antagonized H3R agonist R-α-methylhistamine-induced drinking response in the rat dipsogenia model, improved performance in the rat social recognition model of short-term memory, and showed wake-promoting properties (Raddatz et al., 2012). Recently, a single-blind trial with pitolisant (BF2.649) in 28 adult ADHD patients yielded a progressive improvement in clinical scores. However, the placebo also showed some effect in this trial, so the clinical efficacy is unclear which merits confirmation in a double-blind trial in adults and children (Schwartz, 2011). In addition, MK-0249 (NCT-ID NCT00475735) has completed phase II clinical trials for ADHD but the results are still awaited in public domain (Brioni et al., 2011; Kuhne et al., 2011). PF-03654746 recently completed a phase II trial (NCT00531752) in adult ADHD patients, though no efficacy was observed vs. placebo (Brioni et al., 2011). Likewise, in a randomized clinical study, ADHD-RS-IV score (primary efficacy end point of ADHD) of bavisant, a highly selective, wakefulness-promoting H3R antagonist, though displayed an improved trend, was not statistically significant compared with placebo in ADHD patients (Weisler et al., 2012). These data accumulating from rodent models and clinical settings underpin the idea that modulation of H3R represents a novel therapeutic target for the treatment of ADHD especially due to its wider therapeutic role in CNS and the fact that ADHD is frequently co-morbid with sleep disorders, learning difficulties, substance abuse, anxiety, depression and other neuropsychiatric disorders (Cortese, 2012).

In addition to impaired motor functions and vigilance, ADHD is also associated with impairment of cognitive functions (Bidwell et al., 2011) even though the direct clinical relevance of interventions enhancing cognitive functions in the treatment of ADHD appears limited. Nevertheless, several classes of anti-ADHD drugs have uniformly shown evidence of acute cognitive enhancement and improvement in the core symptoms of ADHD. As described in AD section, a wide array of neurobiological mechanisms have been attributed for the observed pro-cognitive and pro-attentional effects of H3R antagonists in rodent models including H3R antagonists mediated enhancement of neurotransmitter release (Passani et al., 2004; Giannoni et al., 2010) or modulation of electrophysiological activity (Hajos et al., 2008; Andersson et al., 2010) in the hippocampus, induction of immediate early gene expression in brain regions, such as the NAc (Southam et al., 2009) or motor cortex (Bonaventure et al., 2007), that directly regulates locomotor behavior; disruption of dopaminergic activity related to hyperactivity induced by DA agonists, such as apomorphine, cocaine, and methamphetamine (Fox et al., 2005; Ligneau et al., 2007) although paradoxes (Ferrada et al., 2008; Brabant et al., 2009) have also been reported. Recently ciproxifan, H3R antagonist, alleviated hyperactivity and memory impairment in an amyloid-precursor protein transgenic (APPTg2576) mouse model of AD (Bardgett et al., 2011). APP (Tg2576) mice displayed significantly increased LA and longer escape latencies in the swim maze than wild-type mice. In probe trials, ciproxifan-treated APP (Tg2576) mice spent more time near and made more crossings of the previous platform location than did saline-treated APP (Tg2576) mice. Acute ciproxifan treatment also reversed impaired object recognition task in APP (Tg2576) mice (Bardgett et al., 2011). It was hypothesized that the loss of synapses in the hippocampus of APPTg2576 mice possibly caused dysregulation in the NAc and H3R antagonist via increased neurotransmitter release in the hippocampus or by induction of early gene expression in NAc or motor cortex regulated LA.

## Schizophrenia

Schizophrenia, a chronic debilitating neurological syndrome with 0.5–1% prevalence worldwide, is characterized by positive (e.g., hallucination and delusion), negative (e.g., paucity of emotion and motivation), and impaired cognitive symptoms. Though basic knowledge at the level of molecular etiology and psychopathology is inadequate to delineate molecular targets for drug development, dysregulation in DA and other neurotransmitter systems are involved in the development of the disease (Gross and Huber, 2008). Current pharmacotherapy for schizophrenia consists of first generation (FGA) and second generation (SGA) antipsychotics, which mainly act by DA antagonism in CNS mediated mainly by DA D2- receptors but also by D3R and/or D4R, serotonin receptor (5-HTR) subtypes (5-HT2AR/5-HT2CR) and/or via modulation of the glutamatergic system (Sander et al., 2008) which may evoke extrapyramidal (mainly FGAs) and/or serious metabolic disorders (Deng et al., 2010). Due to several adverse effects of antipsychotic therapy, rising therapeutic resistance and inadequate treatment of negative symptoms, H3R antagonist could be a pervasive therapeutic strategy owing to its pro-cognitive property (Ito, 2009).

Histaminergic innervations into the brain areas closely associated with the development of schizophrenia raises the possibility of H3R antagonists influencing its pathophysiology. The clinical relevance of H3R in schizophrenia is bolstered by their high density presence, mostly postsynaptically, in the GABAergic striatal efferent neurons. There they are co-localized with DA D1 and D2 receptors and form heterodimers with the D1 receptor in GABAergic dynorphinergic and with the D2 receptor on GABAergic enkephalinergic neurons, respectively (Pillot et al., 2002a; Ferrada et al., 2008). This intracellular cross-talk results in modified signaling directed by the heterodimer which may underlie the pathogenesis of schizophrenia: e.g., H3-D1 receptor heteromer induces a Gi protein mediated activation of MAPK pathway (Ferrada et al., 2009) whereas H3-D1/D2 heteromers exhibited an antagonistic interaction as H3R agonism negatively modulated D1 or D2 receptor function (Ferrada et al., 2008). H3R antagonists are anticipated to mediate their action by exploiting these functional cross-talks. Ciproxifan, an H3R antagonist, potentiated locomotor hypoactivity and catalepsy induced by haloperidol, a widely used antipsychotic, via enhanced activation of striatopallidal neurons of the indirect movement pathway, upregulation of proneurotensin, proenkephalin, and c-*fos* (Pillot et al., 2002b). Though ciproxifan potentiated haloperidol induced catalepsy, it had no effect on catalepsy when administered alone implicating that no extrapyramidal side effect would result from their therapeutic use when used alone. The same team, a year later, revealed that ciproxifan suppressed the methamphetamine-induced upregulation of striatal neuropeptides like prodynorphin and substance P, the D_1_-receptor-mediated responses; attenuated methamphetamine induced locomotor hyperactivity possibly by inhibition of striatonigral neurons in NAc involved in regulation of locomotor function (Pillot et al., 2003). Thus H3R cross-talk with dopaminergic receptor system has opened a new avenue for designing anti-schizophrenics. Further, BF2.649 activated DA turnover in the PFC but not in striatum, thus possessing a favorable profile similar to SGAs with low liability to induce extrapyramidal symptoms (Ligneau et al., 2007).

As a proof of concept, numerous data has accumulated from schizophrenia cases studied in both rodent models and in human subjects: increased H3R radioligand binding was found in dorsolateral PFC of schizophrenic subjects and bipolar subjects with psychotic symptoms (Jin et al., 2009) and antipsychotic-like profile of H3R antagonists observed in animal models. The latter included improvement of prepulse inhibition (PPI) deficits in rodent schizophrenia models by H3R antagonists/antagonists such as ABT-239 in DBA/2 mice (Fox et al., 2005), pitolisant in Swiss mice (Ligneau et al., 2007), GSK-189254 in Wistar rats (Medhurst et al., 2007), and more recently irdabisant in rats (Raddatz et al., 2012). In DBA/2NCrl mice, CEP-26401 increased PPI of the acoustic startle response alone and also showed synergistic effect when co-administered with risperidone at subefficacious doses (Raddatz et al., 2012). The PPI deficits model the impairment of sensorimotor gating in acoustic startle response in schizophrenic patients. Stimulation of DA activity is critical for attenuation of such deficits induced by amphetamines and other DA agonists and NMDA antagonists. In addition to PPI, antagonism of dopaminergic stimulants (amphetamine, apomorrphine)-induced hyperactivity is important mechanism in pathophysiology of diseases associated with hyperactivity of dopaminergic pathway including schizophrenia and drug abuse. Animal studies demonstrated an antagonism of amphetamine and MK-801 (NMDA R antagonist)-induced motor activation by BF2.649 in mice (Clapham and Kilpatrick, 1994; Ligneau et al., 2007) and attenuation of amphetamine, methamphetamine and apomorphine-induced locomotor stimulation and/or stereotypy by thioperamide and ciproxifan (Clapham and Kilpatrick, 1994; Morisset et al., 2002) in rodent models. While acute amphetamine induced locomotor stimulation is known to represent the euphoric and abusive effects rather than psychosis which is mainly modeled by chronic amphetamine induced locomotor sensitization models (Trujillo et al., 2004), H3R antagonist ciproxifan also reduced LA in methamphetamine induced chronic sensitization model (Motawaj and Arrang, 2011).

Contrary to these, reports ranging from partial or no suppression of apomorphine induced stereotypies (Burban et al., 2010) and no improvement in sensorimotor gating deficits in rats (Southam et al., 2009; Burban et al., 2010) by H3R anatgonists to even exacerbation of hyperactivity (Ferrada et al., 2008; Burban et al., 2010) has also been documented. Cross-talk between H3R and D1/D2 receptor (Ferrada et al., 2008; Burban et al., 2010); interaction between the severity of endpoints (startle magnitude and changes in PPI) used in experimental paradigm that modeled schizophrenia, different levels of PPI among different species and strains (Burban et al., 2010), H3R antagonist used, route administered, specific memory task studied may partially account for the discrepancies (Bardgett et al., 2009) are some of the confounding factors which needs to be taken into account.

Cognitive impairment, a hallmark trait of schizophrenia, is the central determinant of functional outcomes associated with this disease. Although current antipsychotic drugs, mainly targeting the positive psychotic symptoms of the illness, treat psychosis in schizophrenia rather well, their impact on cognitive dysfunction is minimal or largely untreated. In recent years there has been growing interest in developing novel treatments for cognitive deficits in schizophrenia (Ibrahim and Tamminga, 2012). H3R antagonists increased hippocampal gamma (Andersson et al., 2010) and theta oscillation power (Hajos et al., 2008). Deficits of cognition-relevant neuronal oscillations are observed in individuals with schizophrenia which would result in erroneous higher-order cognitive processes, including sensory perception, coherent feature binding, attention, memory, and object representation, that manifest as the positive and negative symptoms of the disease (Shin et al., 2011). Augmentation of DA release in the PFC is key to minimize the negative symptoms and cognitive impairment elicited by antipsychotics. H3R antagonists, enjoy the advantage of enhancing DA release from PFC (Fox et al., 2005; Ligneau et al., 2007; Medhurst et al., 2007), thus may have a sparing effect on prefrontal function over strong DA antagonizing antipsychotics.

Although, inconsistent findings on animal models mimicking schizophrenia does not bring any full proof evidence for antipsychotic properties of H3R antagonists, their waking and pro-cognitive properties uphold their therapeutic interest and can be exploited to adequately address the cognitive impairment central to schizophrenic patients which is resistant to current neuroleptics in hand, hence better clinical outcome. A recent study on ciproxyfan in mice showed a reversal of methamphetamine-induced LA, reversal of downregulation of brain-derived neurotrophic factor (BDNF) and NMDA receptor subunit 1 genes in various regions of mice brain, reinforcing the interest of H3R antagonists in the treatment of cognitive deficits in psychotic patients (Motawaj and Arrang, 2011). Further, the results of clinical studies undertaken by numerous pharmaceutical companies are now eagerly awaited to establish the proof of concept. Pitolisant has completed Phase II of clinical trials showing efficacy in patients suffering from antipsychotic-induced weight gain and hence might be useful for co-treatment (WO2006084833A1). A study designed to assess the pro-cognitive potential of tiprolisant in people with schizophrenia and schizoaffective disorder is currently underway (NCT-ID NCT00690274). A Phase I trial of PF-03654746 as an add-on treatment of cognitive deficits in schizophrenic patients has been completed [87] without publicly disclosed results. H3R antagonists ABT-288, GSK-239512, and MK0249 have recently completed Phase II trials for cognitive impairments in schizophrenia (NCT-ID NCT01077700, NCT01009060, and NCT00506077) although the outcomes of these studies remain undisclosed so far. The findings of ongoing clinical trials in this area will be invaluable in guiding the future course of action.

## Addiction

Addiction is a persistent, compulsive dependence on a behavior or substance. One common mechanism for addiction caused by various drugs of abuse is thought to be the activation of brain's reward circuitry which mainly involves mesolimbic dopaminergic neurons in the ventral tegmental area (VTA) of the midbrain and their targets in limbic forebrain especially NAc (Nestler, 2005). However, other neurotransmitter systems including histamine are known to modulate the psychotropic effects of rewarding drugs [role of histamine reviewed recently by Brabant et al. (2010)]. The fact that histamine also modulates mesolimbic DA transmission suggests that histaminergic drugs may be tried therapeutically in drug addiction (Munzar et al., 2004; Brabant et al., 2010). Though NAc, the main region involved in addiction, receive only weak histaminergic innervations, very high densities of H3Rs are present here. Striatum contains one of the highest densities of H3Rs in the brain (Pollard et al., 1993; Pillot et al., 2002a). Mainly, striatal H3Rs are located postsynaptically in the GABAergic efferent neurons where they are co-localized with D1 and D2 receptors (Ferrada et al., 2008). They are also located presynaptically on dopaminergic terminals in the striatum where they have an inhibitory role on DA release. The central administration of histamine is known to produce a biphasic effect on LA, initially a brief reduction (hypoactivity) followed by a prolonged increase in LA (hyperactivity) (Bristow and Bennett, 1988). The initial hypoactivity is presumed to be due to stimulation of presynaptic H3Rs (heteroreceptors) reducing DA activity and release (Chiavegatto et al., 1998). Thus, histamine is known to have an inhibitory effect on reward by inhibition of mesolimbic dopaminergic system. Histamine can also activate mesolimbic dopaminergic system possibly via presynaptic H3Rs located on DA terminals or postsynaptically on GABAergic neurons in striatum or through H1 Rs located on striatal cholinergic interneurons (reviewed by Brabant et al., 2010). Activation of H3Rs that were found to form heteromers with D2 Rs on GABAergic neurons attenuated stimulant (quinpirole)-induced hyperactivity (Ferrada et al., 2008).

Addictive drugs modulate the histamine levels in the brain. For instance, alcohol affects histamine levels in the brain by modulating histamine synthesis, release and turnover (Zimatkin and Anichtchik, 1999), acute injection of cocaine increases histamine levels and histamine-N-methyl transferase activity in the striatum and NAc (Ito et al., 1997) and opioids (e.g., morphine) increases turnover of neuronal histamine via opioid receptors (Nishibori et al., 1985). On the other hand, histaminergic system can modulate behavioral effects of various drugs of abuse including cocaine, amphetamines, opioids, and alcohol. All addictive drugs stimulate LA (psychomotor stimulant effects). Several studies demonstrate that H1-antagonists produce behavioral and/or neurochemical effect similar to addictive drugs possibly by blocking DA uptake thereby facilitating the activity of mesolimbic dopaminergic system (Brabant et al., 2010; Levin et al., 2011). However, a pharmacokinetic interaction between H1-antagonists (inhibition of CYTP450 CYP2D enzymes) and methamphetamine leading to elevated levels of the latter (Okuda et al., 2004) could not be ruled out. Locomotion activation by amphetamine/methamphetamine and other dopaminergic agonists was attenuated by H3R blockade by thioperamide (Clapham and Kilpatrick, 1994), ciproxifan (Motawaj and Arrang, 2011), ABT-239 (Fox et al., 2005), or BF2.649 (Ligneau et al., 2007) in rodents in acute and chronic behavioral sensitization models. The latter is repeated administration of drug leading to an augmentation of behavioral effects of psychostimulants on re-administration (Celik et al., 2006). However, evidence contradicting the same is also available. For instance, there are cases where H3R antagonists (GSK-207040, Southam et al., 2009 and JNJ-5207852, JNJ-10181457, Komater et al., 2003) did not reverse amphetamine-induced hyperactivity or H3R antagonist thioperamide increased cocaine-induced hyperlocomotion in mice (Brabant et al., 2010). Indeed, the potentiating effects of imidazole-based H3R antagonists like thioperamide, clobenpropit on stimulants could also be due to their inhibitory effects on cytochrome P450 activity as the non-imidazole based antagonist doesn't enhance cocaine-induced stimulant effects (Brabant et al., 2009). Repeated administration of methamphetamine increases HDC activity in striatum and cortex in rats (Ito et al., 1996) and release of histamine in mice (Dai et al., 2004). Consistently, antagonism of H3Rs increases methamphetamine-induced stereotypic hyperactivity in young male mice (Acevedo and Raber, 2011), an effect contradicting the so-called antipsychotic profile of H3R antagonists. There are also reports where thioperamide potentiated the locomotor activation induced by D1 or D2R agonist (Ferrada et al., 2008) suggesting an antagonistic interaction between postsynaptic H3 and D1 or D2 receptors.

Self-administration and conditioned place preference (CPP) paradigms are widely employed to study reinforcing or rewarding effects of drugs of abuse. Histamine has a negative effect on reinforcement because H1-antagonists can act as reinforcers either alone or with other reinforcers like opiates, cocaine, or amphetamine (Brown et al., 2001). Going by this hypothesis, H3R antagonists should prevent reinforcement. Indeed, H3R antagonists (ciproxifan and JNJ-10181457) were found to inhibit the ethanol-evoked CPP whereas H3R agonist immepip did not alter ethanol-induced place preference in male DBA/2J mice. This is further supported by a study where H3 R knock-out (H3RKO) mice did not develop alcohol-induced CPP (Nuutinen et al., 2011). Inhibition of ethanol reward by H3R antagonism implies that H3R might be a noble target to suppress compulsory ethanol seeking (Nuutinen et al., 2011). H3R antagonists also reduced the ethanol drinking in alcohol-preferring (AA) rats (Lintunen et al., 2001; Galici et al., 2011) and blocked rewarding and reinforcing effects of ethanol in DBA/2 mice (Nuutinen et al., 2011). However, they increased acute stimulatory effects of ethanol. The same authors also reported that ciproxifan increased ethanol CPP in mice (Nuutinen et al., 2010) but reduced ethanol-induced locomotor activation in C57BL/6 mice. Consistent to this, thioperamide and clobenpropit increased methamphetamine self-administration and reward possibly through H3 heteroreceptors located on dopaminergic terminals increasing DA release in VTA (Munzar et al., 2004). H3R antagonists also increased cocaine CPP (Brabant et al., 2005) but suppressed LA induced by amphetamine.

Repeated exposure to cocaine, amphetamine or opiates induces phosphorylation and activation of CREB in several reward-related brain regions including NAc whereas alcohol and nicotine reduces CREB phosphorylation in this region. In NAc, psychostimulants induce CREB via activation of DA D1 receptors (Goodman, 2008). Acute administration of ABT239 increased cortical CREB while its continuous infusion normalized reduced cortical CREB phosphorylation in AD transgenic mice (Bitner et al., 2011). In another study, sensitization by methamphetamine-induced reduction of BDNF mRNA in the hippocampus and NMDA R subunit 1 (NR1) mRNA in cerebral cortex, hippocampus and striatum, was reversed by ciproxifan (Motawaj and Arrang, 2011). The effects of H3R antagonists have not been mechanistically evaluated for their effects on CREB phosphorylation or BDNF in brain regions relevant for drug abuse.

## Conclusion

Cognitive dysfunction and motor impairments are the hallmark of multifarious neurodegenerative and/or psychiatric disorders including AD, ADHD, schizophrenia, and drug abuse. The neurochemical basis interlinking these complex behavioral traits in these diseases conditions is complex and it is unclear whether they derive from alterations in a common neuronal circuit. H3R antagonists/inverse agonists, through H3 heteroreceptors, enhance the release of various important central neurotransmitters in brain in addition to histamine and hence can modulate various processes of the CNS including cognition. In addition, postsynaptic H3Rs are present in striatum at GABAergic cell bodies of the medium spiny neurons both on the striatonigral neurons of the direct movement pathway and on the striatopallidal neurons of the indirect movement pathway modulating motor activity (Nuutinen et al., 2011). Locomotor hyperactivity accompanies not only ADHD but also AD and schizophrenia (Viggiano, 2008). Likewise, attentional impairments are observed not only in ADHD but also in Schizophrenia, AD, and drug abuse. It is noticeable that the latter accompanies cognitive and attentional deficits too (Potenza et al., 2011). Such deficits observed in chronic alcohol, cocaine, and methamphetamine abusers is considered to be a particular challenge for treatment-seeking users who require intact cognitive functioning to stop drug abuse. Thus, various cognition enhancing strategies and anti-ADHD drugs have been tried in drug abuse, e.g., atomoxetine, a selective norepinephrine transporter (NET) inhibitor. Similarly, cognitive enhancers are useful in improving attention in ADHD (Bidwell et al., 2011; Levin et al., 2011). Histamine H3R antagonists, though not directly linked to the pathophysiology of disease states, improve cognitive functions and increase alertness, wakefulness, learning, memory without significantly impairing motor functions and thus provides a rationale for their evaluation and research on various neurological conditions where impairment of such functions is paramount especially AD, the most common cause of progressive decline of cognitive functions, and others including ADHD, schizophrenia, epilepsy, narcolepsy, and drug abuse. The broad spectrum of activities of H3R antagonists continues to expand as more and more novel therapeutic roles have been investigated including Parkinson's disease, multiple sclerosis, cerebral ischaemia, depression, etc., and hence identification of potential clinical targets. Nevertheless, many issues such as paucity of clinical data and specific studies evaluating the molecular mechanisms involved in the role of H3R antagonists as well as the conflicting nature of the data gathered so far in pharmacological/biochemical evaluations, remains a challenge that remains to be answered with more rigorous evaluations to develop H3R specific therapeutic agents for human use.

### Conflict of interest statement

The authors declare that the research was conducted in the absence of any commercial or financial relationships that could be construed as a potential conflict of interest.
